# The Efficiency of Combined Capacitive and Resistive Energy Transfer (TECAR) Therapy and Low-Level Laser Therapy (LLLT) in Pain Reduction on Patients With Musculoskeletal Disorders: A Clinical Study

**DOI:** 10.7759/cureus.92670

**Published:** 2025-09-18

**Authors:** Ana-Maria Fatu, Roxana Oana Ciobotaru, Alexia Balta, Alina-Maria Lescai, Nicolae Sarbu, Doina Carina Voinescu, Claudiu Elisei E Tanase

**Affiliations:** 1 Physical Medicine and Rehabilitation, "Dunarea de Jos" University of Galati, Galati, ROU; 2 Cardiology, "Dunarea de Jos" University of Galati, Galati, ROU; 3 Rheumatology, "Dunarea de Jos" University of Galati, Galati, ROU; 4 Medicine, "Dunarea de Jos" University of Galati, Galati, ROU; 5 Radiology, “Dunarea de Jos” University of Galati, Galati, ROU

**Keywords:** low level laser therapy, musculo-skeletal disorders, pain on vas, photobiomodulation, physical medicine and rehabilitation, tecar therapy

## Abstract

Introduction

Musculoskeletal disorders represent a major global burden, being the main cause of global disability. Current management of musculoskeletal pain includes medication (non-steroidal anti-inflammatory drugs, opioids, intra-articular injections), physiotherapy, and surgery. A modern approach in musculoskeletal pain physiotherapy is capacitive and resistive energy transfer (TECAR) therapy associated with low-level laser therapy (LLLT).

Materials and methods

We conducted a prospective, observational, cross-sectional, multicenter clinical study that included a total of 268 patients with clinically and imaging-confirmed musculoskeletal disorders. The collected data were entered and processed using IBM SPSS Statistics for Windows, Version 20 (Released 2011; IBM Corp., Armonk, New York, United States).

Results

The repeated-measures ANOVA revealed a statistically significant and progressive reduction in visual analog scale (VAS) scores across the four time points, demonstrating that the combined TECAR and LASER therapy produced a consistent and clinically relevant improvement in pain. The benefits have been observed across multiple pathologies, including knee osteoarthritis, lumbar and cervical disc herniation, spondylosis, and tendinopathies, with consistent clinical responses across subgroups.

Conclusion

TECAR combined with LLLT represents a non-invasive, effective, and well-tolerated intervention, with the potential to become a central component in the management of chronic musculoskeletal pain. However, further randomized, multicenter studies incorporating functional assessments and long-term follow-up are required to confirm and extend these findings.

## Introduction

According to the World Health Organization (WHO), musculoskeletal disorders represent a major global burden, affecting approximately 1.71 billion people in 2019, being the main cause of global disability [1]. In this category are included more than 150 conditions that affect muscles, bones, joints, and connective tissue, ranging from acute injuries, such as fractures and sprains, to chronic diseases such as osteoarthritis, low back pain, tendinopathy, and fibromyalgia. Of all these, low back pain ranks first in 160 countries, with a higher impact than major depressive disorder or respiratory diseases, as reported by the Global Burden of Disease 2019 study [2]. The number of affected individuals continues to rise due to demographic shifts and lifestyle factors. Early identification, effective management, and integrated preventive interventions (including lifestyle modifications, ergonomics, and access to rehabilitation) can have a significant impact not only on musculoskeletal health but also on the prevention of associated chronic diseases.

Current management of musculoskeletal pain includes medication (non-steroidal anti-inflammatory drugs, opioids, intra-articular injections), physiotherapy, and surgery. Although these approaches can be effective, each presents significant limitations. Pharmacotherapy is associated with gastrointestinal, renal, and cardiovascular adverse effects. Conventional physiotherapy, such as transcutaneous electrical nerve stimulation (TENS), ultrasound, or massage, provides moderate and often transient effects. Surgery, for example, joint replacement in severe osteoarthritis, involves high costs, perioperative risks, and prolonged rehabilitation. This reality has generated increasing interest in non-invasive and well-tolerated methods aimed at reducing pain and improving functionality.

A modern approach in musculoskeletal pain physiotherapy is capacitive and resistive energy transfer (TECAR) therapy. This method employs high-frequency electromagnetic currents, within the range of 0.3-1.2 MHz, which, through deep endothermy, induce important physiological effects such as stimulation of microcirculation, increased oxygen supply at the muscular level, and reduction of edema and local inflammation, thus facilitating lymphatic drainage [3]. By acting on hemoglobin and cellular metabolism, TECAR therapy promotes tissue regeneration processes, while simultaneously decreasing pain intensity [4,5].

According to the International Association for the Study of Pain (IASP), tissue injury activates nociceptor-specialized nerve endings that transmit impulses through Aδ and C fibers to the central nervous system, where they are perceived as pain. Although the fundamental mechanism of TECAR therapy is electromagnetic, its clinical application simultaneously generates micro-tissue vibrations that act on Aβ sensory fibers, inducing presynaptic inhibition at the spinal cord level and thereby blocking the transmission of pain stimuli, an effect explained by the spinal “gate control” theory [6]. Pain relief may also result from a series of indirect physiological mechanisms, including enhanced oxygen and nutrient supply to the tissue, reduction of oxidative stress, and neutralization of pro-nociceptive mediators [7]. An important role is also played by the stimulation of endorphin release, which acts as the body’s natural analgesic [8].

In addition to the analgesic effect, TECAR therapy reduces muscle spasms and contractions, optimizes blood perfusion, and increases tissue oxygenation [8]. In acute inflammatory conditions, the use of thermal procedures is not recommended, as it may exacerbate edema, hemorrhage, and the inflammatory response. Conversely, in chronic inflammatory processes, the application of deep heat facilitates fluid resorption and supports recovery processes [9].

In vitro research has shown that, through the release of growth factors such as TGF-β and bone morphogenetic proteins, this therapy stimulates the proliferation and differentiation of mesenchymal cells into myocytes, chondrocytes, and osteocytes, while also promoting the regeneration of connective tissue structures [10]. TECAR therapy can be applied in both capacitive and resistive modes. The capacitive mode is used for superficial tissues with a high water content, supporting the regeneration of articular cartilage, while the resistive mode is applied to deep tissues with a low water content, promoting bone mineralization [11]. These mechanisms contribute to the accelerated healing of musculoskeletal injuries, including tendon repair, cartilage regeneration, and bone callus formation.

The combination of electromagnetic, bioelectric, and mechanical effects gives this method a complex character, making it an efficient option for modern rehabilitation, capable of supporting the integrated recovery of damaged structures.

Low-level laser therapy (LLLT), also referred to as photobiomodulation therapy (PBM), generally employs radiation with wavelengths between 600 and 1000 nm, with an energy density ranging from 1 to 150 J/cm². Within this spectral range, the best penetration of light in tissues is achieved, as the main chromophores, hemoglobin and melanin, absorb strongly below 600 nm [12]. Thus, wavelengths between 600 and 700 nm are typically used for treatments targeting superficial tissues, while the 780-950 nm range is more suitable for deeper structures [13].

Applied locally or systemically, PBM has documented effects both at the regional and systemic levels, including pain reduction, improved quality of life, decreased muscle spasms, and diminished morning stiffness. Furthermore, research has shown that PBM influences cerebral blood flow, neuronal processes, neuroinflammation, oxidative stress, and neurogenesis, with direct implications for brain network connectivity [14]. By regulating the secretion of hormones such as serotonin and endorphins, LLLT contributes to the reduction of pain signal transmission [15].

Inflammation, sustained by oxidative stress, represents a key element in the pathogenesis of chronic pain. It contributes to the persistence of the inflammatory response and, in the long term, leads to both peripheral and central sensitization, thereby perpetuating pain symptomatology [16]. This phenomenon is considered a major risk factor for persistent chronic pain, being associated with hypersensitization, progression of musculoskeletal disorders, and increased risk of chronic disease. Among involved mediators, there are reactive oxygen species, chemokines, proinflammatory cytokines, transcription factors, and microRNAs [17]. The analgesic effect of laser therapy is attributed primarily to its ability to reduce inflammation, particularly IL-1β and TNF-α mediators, to improve microcirculation, stimulate immune mechanisms, promote regeneration of nerve structures, and enhance β-endorphin release [15]. In addition, low-intensity laser therapy induces vasodilation through endothelium-dependent relaxation of smooth muscle fibers [18]. This process is particularly relevant in the management of joint inflammation, as it facilitates tissue oxygenation and the recruitment of immune cells. Interaction with the vascular endothelium thereby accelerates mechanisms of tissue repair and recovery [19].

## Materials and methods

This was a prospective, observational, cross-sectional, multicenter clinical study conducted at the “St. John” Emergency Clinical Hospital for Children, Galați, and the Reumadiagnostic Clinic, Galați, between October 2023 and June 2025. A total of 268 patients with clinically and imaging-confirmed musculoskeletal disorders were included.

Inclusion criteria

Adults aged over 18 years, reporting pain of at least 4 out of 10 on the visual analogue scale (VAS), who required the application of combined TECAR and LLLT therapy, and who provided written informed consent regarding the use of personal data and adherence to the research protocol, were included.

Exclusion criteria

Patients were excluded if they presented any absolute contraindications for physiotherapy, TECAR, or LLLT: pregnancy, neoplasms, epilepsy, electronic implants, hypertensive crisis, local infections, acute inflammations, decompensated cardiopulmonary disease, acute venous thrombosis, coagulation disorders, hemophilia, photosensitizing medication, or systemic lupus erythematosus.

Treatment protocol

At the Reumadiagnostic Center, LLLT was delivered using the BTL 4000 Premium device in combination with TR-Therapy BTL 6000, applied in dynamic mode. In the Rehabilitation Department of the “St. John” Emergency Clinical Hospital for Children, Galați, the same BTL unit with LLLT was employed together with Care Therapy Phisiomed, applied in static mode. The BTL laser probe emitted infrared radiation at a wavelength of 830 nm, with an output power of 400 W. The parameters used for LLLT are summarized in Table 1. For acute pain, a pulsed emission frequency of 10 Hz was applied, whereas for chronic pain, a continuous frequency was used.

**Table 1:** Parameters of LLLT according to the application site LLLT: Low-level laser therapy

Application area	No of points	Time (minutes)	Energy dose/point	Frequency
Cervical spine	8	8	5J/cm2	10 Hz/continous
Thoracic spine	8	8	5J/cm2	10 Hz/continous
Lumbar spine	8	8	5J/cm2	10 Hz/continous
Shoulder	5	5	4J/cm2	10 Hz/continous
Hand	2	2	3J/cm2	continous
Hip	5	5	6J/cm2	continous
Knee	5	5	5J/cm2	continous
Ankle	5	5	5J/cm2	10 Hz/continous

The radiofrequency therapy protocol was standardized and applied identically to all patients, regardless of pathology or treatment site: 10 minutes in capacitive mode followed by 10 minutes in resistive mode, at low intensity.

Statistical analysis

The collected data were entered and processed using IBM SPSS Statistics for Windows, Version 20 (Released 2011; IBM Corp., Armonk, New York, United States), supplemented with the PROCESS macro v4.2. In the initial phase, a descriptive analysis of the variables was performed to characterize the study population.

Variables that met the criteria for normality were reported as mean ± standard deviation (SD). For ordinal variables (VAS pain scores) that did not follow a normal distribution, the data were summarized using medians and interquartile ranges (IQR). To assess the evolution of parameters across different time points and to capture the cumulative effects of the therapeutic intervention, repeated measures analysis of variance (Repeated Measures ANOVA) was applied. The normality of the VAS data distribution was tested using the One-Sample Kolmogorov-Smirnov test, and the results were used to determine the appropriate statistical tests (parametric or non-parametric). This test was employed to determine whether the data followed a normal (Gaussian) distribution, a prerequisite for the valid application of statistical methods such as the t-test and ANOVA. In cases where the assumption of normality was not met, non-parametric approaches were applied to minimize the risk of misinterpretation. In order to explore potential predictors, multivariate analytical models were developed. Sphericity was evaluated using Mauchly’s test of sphericity. When the assumption was violated (p < 0.05), the Greenhouse-Geisser or Huynh-Feldt corrections were applied to adjust the degrees of freedom. To investigate correlations between continuous variables, Pearson’s coefficient was applied in cases where the data distribution met the assumption of normality. For all statistical analyses, a significance level of p < 0.05 was considered statistically significant.

## Results

The analyzed cohort included 268 patients, of whom 227 (84.7%) were recruited from ReumaDiagnostic Clinic and 41 (15.3%) from “St. John” Emergency Hospital. The age of participants ranged from 19 to 86 years (mean 60.3 ± 11.8), with a predominance of adults and elderly patients. Thus, 3% of the patients were in the 20-40 age group, 42% were between 40 and 60 years old, and 54% were over 60 years old. The sex distribution was unbalanced (75.4% female vs. 24.6% male), suggesting both a higher prevalence of musculoskeletal disorders among women and greater adherence to rehabilitation therapies. Anthropometric assessment showed body weight ranging from 48 to 118 kg (mean 76.7 ± 13.3) and a BMI between 18.82 and 44.41 kg/m² (mean 28.35 ± 4.66), indicating a high frequency of overweight and obesity in the study population.

The distribution of patients according to the pathology for which physiotherapy sessions were performed is presented in Table 2. 

**Table 2:** Physiotherapy-treated conditions

Diagnosis	Frequency	Percent
Wrist osteoarthritis	1	0.4
Ankle (tibio-tarsal) osteoarthritis	2	0.8
Shoulder (scapulohumeral) osteoarthritis	7	2.6
Hip osteoarthritis (coxarthrosis)	12	4.5
Achilles tendinitis	7	2.6
Plantar fasciitis	4	1.5
Knee osteoarthritis (gonarthrosis)	77	28.7
Cervical disc herniation	34	12.7
Lumbar disc herniation	48	17.9
Meniscal knee lesion	9	3.4
Cervical spondylosis	18	6.7
Thoracolumbar spondylosis	12	4.5
Lumbar spondylosis	16	6.0
Supraspinatus tendinopathy	21	7.9
Total	268	100.0

The analysis shows that knee osteoarthritis (gonarthrosis) accounts for the largest proportion, representing 28.7% of all cases. This is followed by lumbar disc herniation (17.9%) and cervical disc herniation (12.7%). Other diagnoses with notable frequencies included cervical spondylosis (6.7%), lumbar spondylosis (6%) and hip osteoarthritis (coxarthrosis) (4.5%). Overall, the distribution confirms the predominance of degenerative diseases affecting large joints and the spine, reflecting the profile of the majority of patients requiring rehabilitation treatment.

The typology of diseases treated by physiotherapy indicates that degenerative joint disorders constitute the dominant category, summing 53% of cases. These are followed by discopathies, representing 31%, and tendinopathies, with a proportion of 11%. The axial region (spinal column) was the most frequently treated site with combined TECAR/LLLT therapy, representing 48% of all cases analyzed. In descending order of frequency, these were followed by large joints of the lower limbs (37%), upper limbs (11%), and, with a lower proportion, small joints of the lower limbs (4%). Degenerative processes involving large joints and spinal pathologies are therefore the main reasons for patients’ referral to rehabilitation services, underscoring both the substantial impact of these conditions on quality of life and the frequent need for targeted therapeutic interventions.

Pain assessment was performed using the VAS at four distinct time points: before treatment (VAS_ini) and subsequently on days 1, 5, and 10 (VAS_z1, VAS_z5, VAS_z10). Given the ordinal nature of the variables and their deviation from normal distribution, the results were reported using medians and suitable interquartile ranges, in order to accurately capture the dynamics of symptoms throughout the therapeutic intervention.

The VAS is one of the most widely used tools for quantifying pain intensity, owing to its simplicity and broad clinical applicability. Patients are asked to mark the perceived level of pain on a 10-cm continuous line, anchored by “no pain” (0) at one end and “worst imaginable pain” (10) at the other. Interpretation of the scores relies on clinical thresholds: 0 - no pain, 1-3 - mild pain, 4-6 - moderate pain, 7-10 - severe pain. This classification enables both the monitoring of symptom evolution over time and the evaluation of therapeutic effectiveness. In clinical research, a reduction of at least 2 points on the VAS or a decrease of 30% compared to baseline is generally considered a clinically significant response.

According to Table 3, the Kolmogorov-Smirnov test indicated that the distributions of VAS scores were consistent with the assumption of normality at all assessment points (p > 0.05): baseline p = 0.459, day 1 p = 0.295, day 5 p = 0.052, and day 10 p = 0.098.

**Table 3:** Kolmogorov-Smirnov Test for assessing the normality of VAS (visual analogue scale) variables a Test distribution is normal. b Calculated from data.

One-Sample Kolmogorov-Smirnov Test		VAS Score initial (mm)	VAS Score After Therapies – Day 1 (mm)	VAS Score After Therapies – Day 5 (mm)	VAS Score After Therapies – Day 10 (mm)
N		268	268	268	268
Normal Parameters^a,b^	Mean	65.12	52.19	37.21	21.85
	Std. Deviation	20.894	19.934	17.816	14.259
Most Extreme Differences	Absolute	0.052	0.06	0.083	0.075
	Positive	0.052	0.06	0.083	0.075
	Negative	-0.049	-0.042	-0.043	-0.063
Kolmogorov-Smirnov Z		0.854	0.977	1.352	1.227
Asymp. Sig. (2-tailed)		0.459	0.295	0.052	0.098

Since the criterion of statistical normality was met, comparative analysis of VAS means could be performed using parametric tests, providing a robust basis for interpretation of investigated treatments' efficiency.

The analysis of VAS score variation across the four evaluation points requires a statistical approach adapted to the dependent nature of the data. Repeated measures analysis of variance (ANOVA) is considered the gold standard procedure in such cases, as it allows the detection of overall differences across time points and the assessment of both main effects and interactions.

VAS scores recorded at the four time points (baseline, day 1, day 5, and day 10) served as the dependent variable. The within-subject factor “Time of assessment” comprised four levels, allowing for the evaluation of pain dynamics over time. The assumption of sphericity was tested using Mauchly’s test; when this assumption was violated, Greenhouse-Geisser or Huynh-Feldt corrections were applied to adjust the degrees of freedom. The level of statistical significance was set at p < 0.05.

Table 4 shows a consistent decrease in the mean VAS score during the course of combined TECAR and LASER therapy. The initial mean value was 65.12 points, decreasing to 52.19 after the first day of treatment, to 37.21 on day 5, and reaching 21.85 at the end of day 10. The downward trend of mean values, accompanied by a reduction in standard deviation, indicates a progressive and consistent improvement in pain within the analyzed cohort.

**Table 4:** Descriptive statistics of VAS scores across four time points VAS: Visual analogue scale

Descriptive Statistics	Mean	Std. Deviation	N
VAS Score initial (mm)	65.12	20.894	268
VAS Score After Therapies – Day 1 (mm)	52.19	19.934	268
VAS Score After Therapies – Day 5 (mm)	37.21	17.816	268
VAS Score After Therapies – Day 10 (mm)	21.85	14.259	268

The downward trend in mean values, accompanied by a reduction in standard deviation, indicates a progressive and consistent improvement in pain within the analyzed cohort. According to Table 5, the factor “Time” exerts an extremely strong influence on VAS scores, with the following test results: Pillai’s Trace = 0.893, F(3, 265) = 734.964, p < 0.001.

**Table 5:** Multivariate tests regarding the effect of time on VAS scores a Exact statistic b Computed using alpha = 0.05 VAS: Visual analogue scale

Effect		Value	F	Hypothesis df	Error df	Sig.	Partial Eta Squared	Noncent. Parameter	Observed Power^b^
Time	Pillai's Trace	0.893	734.964^a^	3	265	0.000	0.893	2204.893	1
	Wilks' Lambda	0.107	734.964^a^	3	265	0.000	0.893	2204.893	1
	Hotelling's Trace	8.32	734.964^a^	3	265	0.000	0.893	2204.893	1
	Roy's Largest Root	8.32	734.964^a^	3	265	0.000	0.893	2204.893	1

The Partial Eta Squared value of 0.893 indicates that 89.3% of the variability in VAS scores can be attributed to differences between assessment time points, confirming the major impact of the combined TECAR and LASER therapy in reducing pain.

The results of Mauchly’s test indicate in Table 6 that the assumption of sphericity was violated (W = 0.701, χ²(5) = 94.462, p < 0.001), confirming that the variances of the differences between measurement points were not uniform.

**Table 6:** Mauchly’s test of sphericity Tests the null hypothesis that the error covariance matrix of the orthonormalized transformed dependent variables is proportional to an identity matrix. a May be used to adjust the degrees of freedom for the averaged tests of significance. Corrected tests are displayed in the Tests of Within-Subjects Effects table.

Within Subjects Effect	Mauchly's W	Approx. Chi-Square	df	Sig.	Epsilon^a^		
					Greenhouse-Geisser	Huynh-Feldt	Lower-bound
Time	0.701	94.462	5	0.000	0.852	0.861	0.333

Since this assumption is a prerequisite for the correct application of ANOVA, the violation of sphericity requires the use of adjustments. Consequently, the degrees of freedom were corrected using the Greenhouse-Geisser or Huynh-Feldt methods, in order to ensure the statistical validity of the tests and to minimize the risk of Type I errors.

The analysis presented in Table 7 demonstrates a significant impact of the variable “Time” on VAS scores, which remained consistent even after applying the Greenhouse-Geisser correction (F(2.852; 757.864) = 1024.646, p < 0.001). The magnitude of the effect, reflected by a Partial Eta Squared value of 0.793, indicates that nearly 80% of the variability in scores is explained by differences between successive evaluation points.

**Table 7:** Effect of time on VAS scores within-subjects effects–repeated measures ANOVA a Computed using alpha = 0.05 VAS: Visual analogue scale

Source		Type III Sum of Squares	df	Mean Square	F	Sig.	Partial Eta Squared	Noncent. Parameter	Observed Power^a^
Time	Sphericity Assumed	281309.674	3	93769.89	1024.646	0.000	0.793	3073.937	1
	Greenhouse-Geisser	281309.674	3	110103.9	1024.646	0.000	0.793	2617.915	1
	Huynh-Feldt	281309.674	3	108961.7	1024.646	0.000	0.793	2645.359	1
	Lower-bound	281309.674	1	281309.7	1024.646	0.000	0.793	1024.646	1
Error(Time)	Sphericity Assumed	73303.076	801	91.514					
	Greenhouse-Geisser	73303.076	682	107.456					
	Huynh-Feldt	73303.076	689	106.341					
	Lower-bound	73303.076	267	274.543					

The results shown in Table 8 highlight a very strong linear trend in the reduction of VAS scores (F(1, 267) = 2218.636, p < 0.001, η²p = 0.893), confirming a consistent decrease in pain intensity over time and a treatment effect of high magnitude. In practical terms, pain levels decreased steadily throughout the intervention, supporting the effectiveness of the applied protocol across the entire study cohort.

**Table 8:** Tests of within-subjects contrast with repeated measures ANOVA a Computed using alpha = 0.05

Source	Time	Type III Sum of Squares	df	Mean Square	F	Sig.	Partial Eta Squared	Noncent. Parameter	Observed Power^a^
Time	Linear	280880.1	1	280880.1	2218.636	0.000	0.893	2218.636	1
	Quadratic	392.9	1	392.9	4.823	0.029	0.018	4.823	0.59
	Cubic	36.6	1	36.6	0.551	0.459	0.002	0.551	0.115
Error(Time)	Linear	33802.3	267	126.6					
	Quadratic	21752.3	267	81.5					
	Cubic	17748.4	267	66.5					

In addition to this linear trend, a significant quadratic component was also identified (F(1, 267) = 4.823, p = 0.029, η²p = 0.018), suggesting that the rate of symptom reduction was not uniform. Pain relief was more pronounced during the initial days of therapy, followed by a gradual attenuation of the reduction rate in subsequent stages. From a clinical perspective, this dynamic indicates that the initial treatment response is both rapid and consistent, while continuation of the sessions ensures the consolidation and maintenance of therapeutic benefits.

The data presented in Table 9 highlight the statistical significance of the intercept (F(1, 267) = 1929.732, p < 0.001), accompanied by a partial Eta squared coefficient of 0.878.

**Table 9:** Between-Subjects Effects in Repeated Measures ANOVA a Computed using alpha = 0.05

Source	Type III Sum of Squares	df	Mean Square	F	Sig.	Partial Eta Squared	Noncent. Parameter	Observed Power^a^
Intercept	2084289.5	1	2084290	1929.732	0.000	0.878	1929.732	1
Error	288384.73	267	1080.093					

The overall mean of VAS scores deviates significantly from zero and accounts for 87.8% of the total observed variability.

The efficiency of therapeutic interventions may vary depending on the anatomical region involved, the differences being determined by factors like specific biomechanic loading, vascularization, and the degree of joint complexity. These structural and functional differences distinguish areas such as the spine, weight-bearing joints (knee, hip), and peripheral joints (ankle, hand). 

Table 10 presents the analysis conducted to test the hypothesis that the anatomical location of the pathology may influence the effectiveness of the combined TECAR-LASER physiotherapeutic treatment in reducing pain. For this purpose, the pain scores were compared using the VAS as the primary assessment tool.

**Table 10:** Descriptive statistics of the absolute pain difference (D_VAS = VAS_initial – VAS_day10) by anatomical location treated

Absolute Pain Difference	N	Mean	Std. Deviation	Std. Error	95% Confidence Interval for Mean		Min	Max
					Lower Bound	Upper Bound		
Upper limbs	29	45.31	15.557	2.889	39.39	51.23	23	84
Lower limbs – small joints	12	45.17	14.141	4.082	36.18	54.15	20	71
Lower limbs – large joints (knee and hip)	99	42.39	14.52	1.459	39.5	45.29	11	76
Axial region (spine)	128	43.3	16.538	1.462	40.41	46.2	8	94
Total	268	43.27	15.556	0.95	41.4	45.14	8	94

The analysis of the data presented in Table 10 indicates that the average pain reduction is relatively similar across the anatomical regions assessed. The mean values were 45.31 for the upper limbs, 45.17 for the lower limbs - small joints, 42.39 for the lower limbs - large joints, and 43.30 for the axial region. The confidence intervals overlap considerably, suggesting that the observed mean differences of approximately 2-3 points are small relative to the within-group variability (SD ~14-17). Additionally, the unequal sample sizes across groups (e.g., N = 12 for “small joints” vs. N = 128 for “axial region”) caution is required interpreting these descriptive differences, as they may affect the reliability and generalizability of the results.

Based on Levene's test for homogeneity of variances, there is insufficient evidence to reject the null hypothesis of equal variances (Levene = 0.534; p = 0.659). The comparative analysis between groups, presented in Table 11, can be extended using a classic one-way ANOVA. 

**Table 11:** Univariate ANOVA for absolute difference in pain (D_VAS = VAS_ini – VAS_z10) according to the anatomical location

	Sum of Squares	df	Mean Square	F	Sig.
Between Groups	240.03	3	80.01	0.328	0.805
Within Groups	64374.63	264	243.843		
Total	64614.66	267			

The results presented in Table 11 show no significant differences between anatomical regions regarding pain reduction after TECAR-LASER treatment: F(3, 264) = 0.328, p = 0.805. The effect size is minimal (η² ≈ 0.004), indicating that the anatomical location accounts for less than 1% of the variability in pain reduction.

## Discussion

Our results demonstrate a substantial and sustained reduction in pain intensity (VAS) over the 10-day course of combined TECAR + LLLT therapy, with a very large overall effect size. These findings are consistent with existing evidence positioning capacitive-resistive energy transfer diathermy (TECAR/CRET) as a feasible analgesic option in musculoskeletal disorders, while adding an important element: the association with photobiomodulation (LLLT) may enhance the clinical response. In the current literature, the quality of evidence regarding electromagnetic diathermy remains heterogeneous; however, recent systematic reviews indicate beneficial effects on both pain and function, noting that many studies are small and/or at risk of bias, which warrants cautious interpretation [20].

Regarding TECAR, two randomized studies are worth highlighting. Notarnicola et al. compared TECAR therapy with laser therapy (applied as monotherapy) in patients with low back pain, reporting significantly greater improvements in the TECAR group at 1-2 months post-treatment (VAS and disability), supporting the clinical value of capacitive-resistive energy transfer in nonspecific low back pain [21]. In another RCT, Kasimis et al. demonstrated that the addition of TECAR to manual therapy produced a significant supplementary benefit compared with manual therapy alone [22]. Furthermore, in a multicenter RCT, RF diathermy was compared with ultrasound therapy in patients with low back pain; both interventions improved pain and function, but RF achieved a higher proportion of “pain responders” at 12 weeks, suggesting a potential clinical advantage of deep heating via radiofrequency [23].

For LLLT, high-quality evidence is more robust in certain indications. The meta-analysis by Stausholm et al. (22 RCTs, n = 1063) demonstrated a significant reduction in pain and disability in knee osteoarthritis, with greater effects observed when the WALT-recommended dosages were applied (e.g., 4-8 J at 785-860 nm or 1-3 J at 904 nm per point), and no adverse events were reported [24]. In lower limb tendinopathies and plantar fasciitis, a meta-analysis by Naterstad et al. confirmed significant pain reductions and functional improvements in the short- and medium-term [25]. In chronic nonspecific low back pain, a 2015 review and meta-analysis found a significant analgesic effect, although the impact on function was less clear; in cervical pain, earlier syntheses indicated benefits that persisted for up to 22 weeks after treatment completion [26].

The data support the hypothesis that the effects of TECAR (deep heating, increased blood flow and oxygenation, modulation of peripheral excitability) and LLLT (inflammatory and mitochondrial modulation) are complementary, providing a solid biological rationale for their combined use. The literature on the concomitant use of TECAR and LLLT is limited. Our study provides one of the first pieces of evidence in a large cohort (268 patients), supporting the hypothesis that the effects of the two therapies are synergistic: TECAR prepares the tissue (by increasing blood flow and oxygenation), while LLLT modulates inflammation and pain at the cellular level.

Clinical implications

Rapid Onset

Both TECAR and LLLT can produce clinically detectable pain relief after the first session, making them valuable for acute exacerbations.

Consistency

They demonstrated efficacy across multiple musculoskeletal conditions, including osteoarthritis, discopathies, and tendinopathies.

Integration

They are suitable for incorporation into standard rehabilitation protocols alongside exercise therapy, with evidence of additive effects.

Cost-effectiveness

By reducing analgesic requirements and chronicity risk, combined TECAR+LLLT has the potential to lower healthcare costs.

Limitations

This study has several limitations that must be taken into account when interpreting the results. The lack of randomization and a control group, as well as the absence of subgroup analyses by age and comorbidities, may reduce the validity of the conclusions and do not allow the exclusion of placebo effects or other nonspecific factors, such as the therapist-patient relationship or patient expectations. Pain assessment relied solely on the VAS scale, which, while widely used and validated, can be influenced by factors such as age, educational level, or cognitive impairments, and does not fully capture the clinical impact of the intervention. The inclusion of additional tools, such as functional scores (WOMAC, Oswestry, DASH) and quality-of-life questionnaires (SF-36), would have provided a more comprehensive evaluation of treatment outcomes. The only 10 days follow-up short period does not provide information regarding the sustainability of benefits over the medium and long term. Since the study population consisted of chronic patients who typically return approximately every six months for rehabilitation treatment, future research has the opportunity to extend follow-up to better assess the persistence of therapeutic effects.

To strengthen the scientific evidence, future studies should employ a randomized design with control groups, including subgroup analyses, integrate functional and quality-of-life assessments, and implement long-term monitoring. These methodological improvements would enhance both the internal and external validity of the findings and support the development of robust clinical recommendations.

## Conclusions

Combined TECAR and LLLT therapy proved to be a non-invasive, safe, and well-tolerated intervention, capable of producing a rapid and significant reduction in pain across various musculoskeletal disorders. The findings suggest a potential synergistic effect of the two modalities and support their integration into rehabilitation protocols, with the prospect of reducing the clinical and socioeconomic burden of these conditions. Nevertheless, further randomized, multicenter studies with functional outcomes and long-term follow-up are required to confirm and consolidate these results.
